# A Novel Hybrid Logic-ODE Modeling Approach to Overcome Knowledge Gaps

**DOI:** 10.3389/fmolb.2021.760077

**Published:** 2021-12-20

**Authors:** Gianluca Selvaggio, Serena Cristellon, Luca Marchetti

**Affiliations:** ^1^ Piazza Manifattura, Fondazione The Microsoft Research-University of Trento Centre for Computational and Systems Biology (COSBI), Rovereto, Italy; ^2^ Department of Mathematics, University of Trento, Trento, Italy; ^3^ Department of Cellular, Computational and Integrative Biology (CIBIO), University of Trento, Trento, Italy

**Keywords:** hybrid modeling, logic modeling, ordinary differential equations (ODEs), computational systems biology, simulation algorithms

## Abstract

Mathematical modeling allows using different formalisms to describe, investigate, and understand biological processes. However, despite the advent of high-throughput experimental techniques, quantitative information is still a challenge when looking for data to calibrate model parameters. Furthermore, quantitative formalisms must cope with stiffness and tractability problems, more so if used to describe multicellular systems. On the other hand, qualitative models may lack the proper granularity to describe the underlying kinetic processes. We propose a hybrid modeling approach that integrates ordinary differential equations and logical formalism to describe distinct biological layers and their communication. We focused on a multicellular system as a case study by applying the hybrid formalism to the well-known Delta-Notch signaling pathway. We used a differential equation model to describe the intracellular pathways while the cell–cell interactions were defined by logic rules. The hybrid approach herein employed allows us to combine the pros of different modeling techniques by overcoming the lack of quantitative information with a qualitative description that discretizes activation and inhibition processes, thus avoiding complexity.

## Introduction

Computational models have become a cornerstone of modern biology as a tool for data interpretation and serving in parallel with experimental techniques to disentangle process complexity (Markowetz, 2017). Depending on the available information and the addressed questions, we can model the processes with different approaches, in function of model granularity and abstraction level (Ideker and Lauffenburger, 2003). Biological processes for which are available high-throughput omics data can be described using interaction networks (Danaher et al., 2014; Hawe et al., 2019); these can be integrated with other experimental evidences (such as knock-out experiments) to provide directed graphs (Gross et al., 2019). By increasing the biological knowledge of the processes involved, providing the sign of the interactions, we can obtain a regulatory graph that together with a set of rules for each component defines a logic model (Abou-Jaoudé et al., 2016).

The logic formalism is the simplest way to model interactions among entities, in a parameter-free fashion, and has been used since the 1970s to qualitatively describe biological pathways (Kauffman, 1969), and intracellular and intercellular signaling networks (Gonzalez et al., 2008; Morris et al., 2010) up to collective cell behaviour (Varela et al., 2018). In the case that the pathways and their components are thoroughly characterized, ordinary and partial differential equations (ODEs and PDEs) can be used instead (Aldridge et al., 2006). If the number of molecules involved in the processes does not meet the Continuum hypothesis requirements, then the stochastic methods allow to overcome the problem (Simoni et al., 2019).

Selecting the appropriate mathematical formalism to describe the biology is thus a trade-off between the a priori available knowledge (e.g., parameters, concentrations etc.) and the required granularity to address the biological problem.

Another important aspect to consider in systems biology is the interplay between different biological layers, as most models focus on a single scale. Progresses have been made in implementing hierarchical representations to study how local variations may affect the dynamics at other levels. An example of this approach is the work of Uluseker et al. (2018) in which the authors built an ODE model that integrates in a holistic framework the glucose homeostasis together with other regulatory hormones at different levels: gut, liver, and adipose tissue. However, models that span over different levels (e.g., from subcellular to tissue) are difficult to parametrize and implement. Model combination (Palsson et al., 2013) can be a solution but it is a challenging task due to the non-modular implementation of these mathematical frameworks, and goes beyond simple coupling of the equations.

The technical difficulties of model integration, arising from the different modeling formalisms, have been tackled by recasting the mathematical descriptions to a single approach. Ryll et al. (2014), with their model of hormonal regulation of glucose homeostasis, proposed a strategy to integrate a logic model of signaling network with an ODE model of metabolic processes: the Boolean representation was converted into a set of logic-based ODEs. The integration required a calibration step in which the added parameters and the missing ones of the kinetic model were fitted to experimental data.

As outlined in Uluseker et al. (2018), biological phenomena interlay different abstraction levels, where interconnected modules form complex collective behaviour. Hierarchical models, as mentioned previously, are used to provide a structured holistic representation of complex biological systems. Single models communicate, through feedbacks, at the systemic level producing the macroscopic behavior. Often, for these interactions, only limited knowledge or qualitative measurements are available and thus a complete ODE description is hindered. Here, we propose an integrative approach that leverages on different formalisms (ODE and logic), with a fine-grained ODE representation of the bottom layer to properly describe the variable dynamics, and the logic formalism to represent in a coarse-grained fashion the regulative interactions. This approach does not require a model re-parametrization or recasting to a common description thus enabling model reuse.

Although the modeling approach is the focus of the present work, we decided to convey our strategy by presenting a case study: the Delta-Notch signaling pathway.

Delta-Notch signaling is among the most conserved pathways in tissue development based on the negative-feedback loop between the two elements (Artavanis-Tsakonas et al., 1999). Upon Delta ligand binding to the Notch receptor of another cell, a response is triggered leading the receiving cell to repress Delta, governing fate selection. Several examples of Delta-Notch salt-and-pepper patterning are present in nature like in *Drosophila* (Renaud and Simpson, 2001; De Joussineau et al., 2003), as well as in the mouse inner ear (Hartman et al., 2010), and mouse and zebrafish retina (Del Bene et al., 2008).

One of the first models investigating the Delta-Notch signaling pathway, developed by Collier et al. (1996), used the ODE formalism to qualitatively describe the dynamics of active Notch and Delta between adjacent cells. The input of a generic cell was modeled as the average of all neighbor Deltas. The intracellular Notch activation and consequent fate decision were described using a phenomenological Hill function. Agrawal et al. (2009) adopted a fine-grained approach describing with an ODE system the intracellular processes of cleavage, transcription, translation, transport, and degradation, after Notch activation. The work focused on the analysis of the single cell fate decision, rather than the pattern formation, highlighting the possibility of a phenotypic switch from bistable system to oscillatory, by tuning a single parameter. Mjolsness et al. (1991) and Marnellos and Mjolsness (1998) modeled neuroblasts and sensory organ precursor cell differentiation in *Drosophila*, as nodes in a recurrent neural network. Cells are represented as discrete entities, which can interact with neighbors. A minimal two-gene network was used, allowing interaction with other gene products from within the same cell or from neighboring ones. Varela et al. (2018) developed a 2D logical model of lateral inhibition, using the software Epilog. Each cell of the discrete tissue contains a two-component logic model that responds to the input coming from the neighboring cells. Both Varela et al. and Marnellos et al. approaches allow to simulate pattern formation at the tissue level without in depth knowledge of the kinetic parameters or species concentrations. Also, agent-based models (ABM) have been employed to address how complex behaviors arise from the cell–cell interactions (Reynolds et al., 2019) or cell–environment interaction (Yu and Bagheri, 2020). In particular, Reynolds et al. (2019) developed an ABM that recreates Delta-Notch patterns using for each agent a set of rules that define the increment of each species, thus providing a more abstract view.

The hybrid strategy we propose defines a semi-quantitative framework optimal to simulate tissue level dynamics with fixed interacting cells. The implementation, at the lower level, of an ODE-based model generates a quantitative time that allows to better appreciate the grid evolution. Complex behavior and spatial effects can be implemented including in the logical rules more than the first line of neighbors and by changing the geometry of the grid (i.e., cylindrical, toroidal etc.).

### Materials and Methods

The intracellular signaling cascade model parameterization is provided in Supplementary Material S1. All computations were implemented and performed in MATLAB (R2019b); simulations were performed with an Intel Core i7-8700T processor, CPU 2.40 GHz and installed RAM 16.00 GB. Numerical integration of the ODE system was made using the ODE solver ode15s.

## Results

### Hybrid Modeling Approach

In this work, we propose a hybrid modeling approach to describe complex biological phenomena where the lack of kinetic parameters, species concentrations, or mechanistic knowledge hinders a complete ODE description.

**FIGURE 1:**
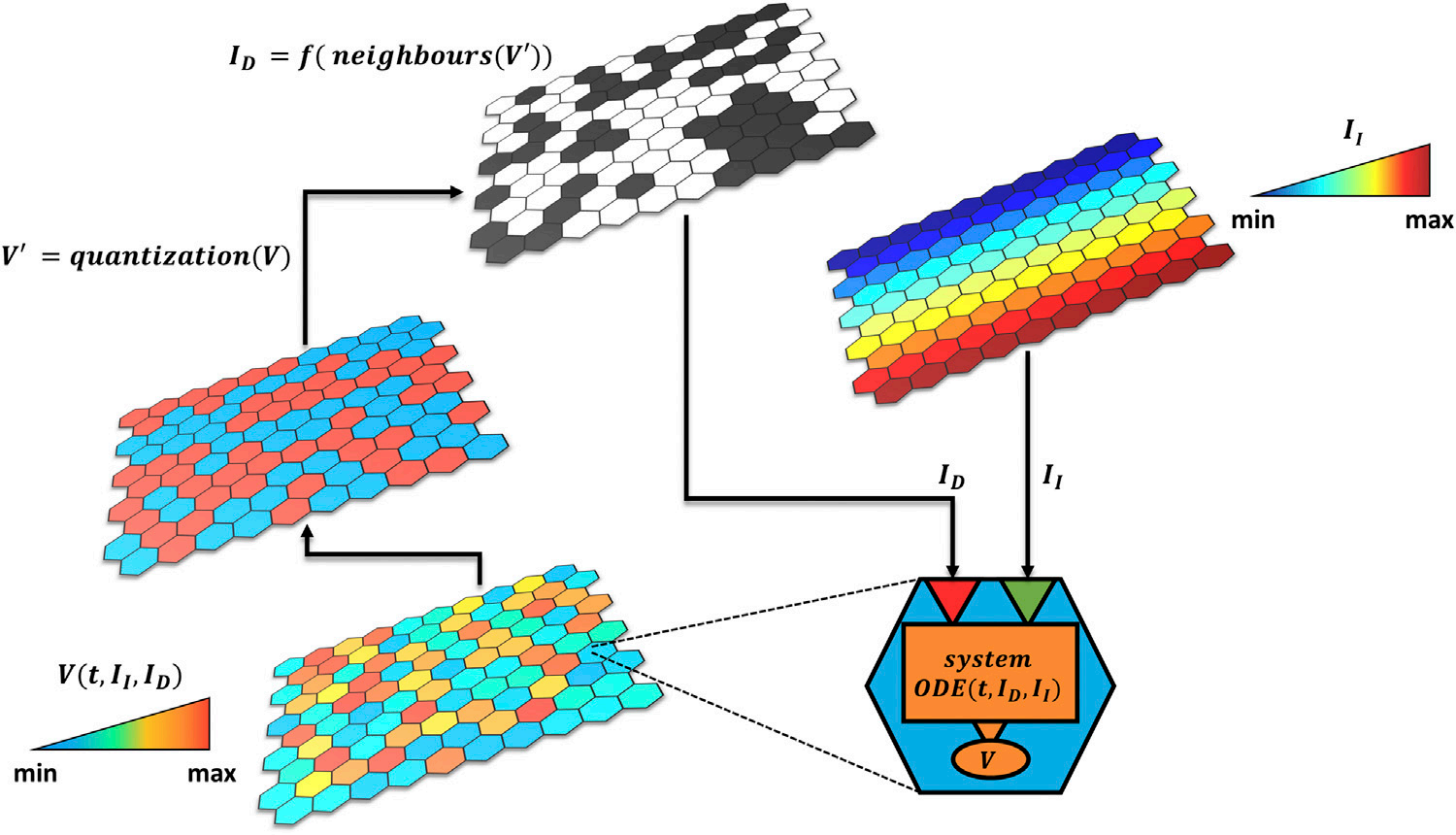
Hybrid model approach. The tissue is composed by a grid of quantitative modules described by an ODE system depending on two types of inputs $I_{D}$ and $I_{I}$ , respectively dependent and independent from the other modules. The former is usually cast as a function of the internal variable ( V ) of neighboring cells. Thresholding the Vs of all cells will generate a logical matrix ( V' ), which is then used to compute $I_{D}$ by applying a logical rule f . $I_{I}$ identifies instead those inputs that are only dependent from the spatial position.

The model is built hierarchically in a bottom-up fashion (Figure 1). At the lower level, there is a matrix of quantitative single modules (i.e., signaling pathways, cells) described by a system of ordinary differential equations characterized by a set of variables. Each module can receive two types of inputs: independent (I_I_) or dependent (I_D_) from the other modules. The latter is a logical variable (Boolean or multivalued) which describes the interactions between the single modules or environmental feedbacks (i.e., pathway crosstalk, extracellular signaling) and encodes through a logical rule the contribution of the neighboring modules’ output variables (*V*). The independent input, $I_{I}$ , is used instead to portrait those signals that are only position dependent, as diffusive molecules or other environmental cues.

**FIGURE 2:**
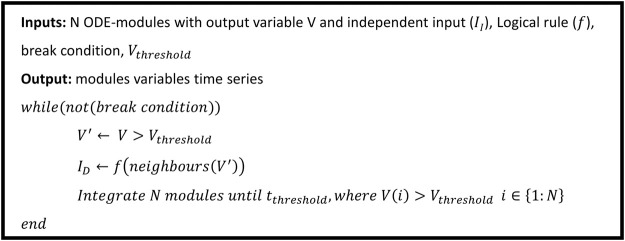
Hybrid model simulation pseudo-code, an extended version of the code is available in Supplementary Material S1.

The hybrid approach we propose thus puts into communication the two layers (intracellular and tissue) and can be simulated as illustrated by the pseudo-code of Figure 2.

While $I_{I}$ is uniquely defined by the cell position, $I_{D}$ is locally defined by the variable *V* of the neighboring modules. To define each module’s $I_{D}$ , we first threshold V for all the modules, generating the logic matrix V' ; we compute $I_{D}$ by applying the logical rule f on V' , considering a specific neighborhood of the module of the grid in exam. Given the planar representation and the finite number of grid elements, border cells may have a lower number of neighbors when compared with the others; it is thus important to define boundary conditions to mitigate artifact effects. Possible strategies that we also apply in the context of the case study, are cylindrical conditions, where two borders of the grid are put in contact by a single fold of the grid or toroidal conditions, where a double fold of the grid put in pairwise contact the grid borders. The modules are then integrated until the variable V crosses the quantization threshold ( $t_{threshold}$ ).

The process is then repeated until the break condition is met; this can be grid equilibrium, maximum allowed simulation time, or other ad hoc constrains.

## Case Study: Delta-Notch

Delta-Notch is a highly conserved cell–cell communication pathway present in most animals; it allows cells to select different fates based upon the neighborhood consensus. In *Drosophila*, during the neuronal development phase, cellular differentiation gives rise to salt-and-pepper patterns with cells either reaching neuronal fate or not (Campos-Ortega, 1995; Bertrand et al., 2002). The phenomenon of lateral inhibition among adjacent cells, mediated by Delta-Notch signaling pathway, has a major role in this kind of pattern formation. Depending on the interconnectivity of the network, different patterns can arise, and since cells are observed to extend protrusions, even non-adjacent cells can interact (Renaud and Simpson, 2001; Hadjivasiliou et al., 2016).

In the following sections, we will describe how we developed and integrated the intracellular ODE model and the intercellular logic model.

### Intracellular Signaling Cascade

**FIGURE 3:**
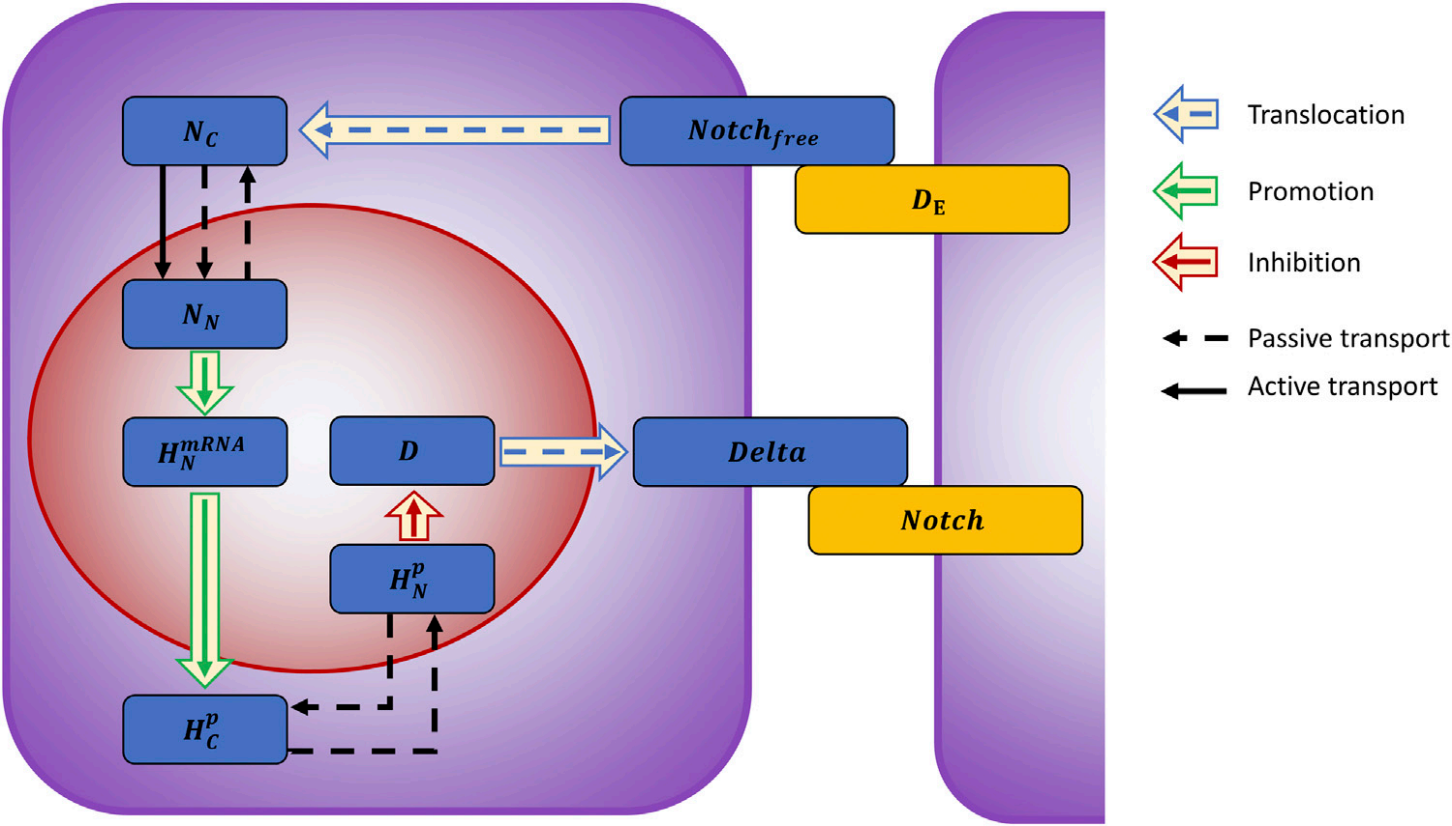
Graphical representation of the model describing the Delta-Notch pathway. Upon binding to the Delta ligand ( $D_{E}$ ) from another cell (yellow rectangle) the Notch receptor releases its intracellular domain in the cytoplasm ( $N_{C}$ ). After entering the nucleus, either due to passive or active transport, Notch ( $N_{N}$ ) acts as transcription factor inducing the expression of Hes mRNA ( $H_{N}^{mRNA}$ ) and its translation into Hes protein ( $H_{C}^{p}$ ). $H_{C}^{p}$ migrates into the nucleus ( $H_{N}^{p}$ ) where it acts as repressor of Delta expression (D) leading to a decrease in Delta protein at the membrane. Coupling this system with its counterpart in a neighbor cell will lead to having a Delta^+^ cell and a Delta^−^ cell, respectively expressing or not the protein. The model consists of two cell compartments: nuclear (red) and cytoplasmatic (violet).

We developed a mathematical model describing the Notch intracellular pathway, building upon the computational work of Agrawal et al. (2009) (for details see Supplementary Material S1). A set of ordinary differential equations quantitatively trace the different components involved in the processes, following the Delta-Notch binding on the outer membrane (Figure 3).

The cell is modeled with two compartments: cytoplasm (volume $V_{C}$ ) and nucleus (volume $V_{N}$ ). Every cell receives as external input the ligand Delta ( $D_{E}$ ) from other cells; we considered it binary: either present ( $D_{E}=D_{E}^{max}$ ) or not ( $D_{E}=0$ ). Upon binding of $D_{E}$ with free Notch ( $Notch_{free}$ ), Notch intracellular domain is cleaved and released in the cytoplasm ( $N_{C}$ ) with a rate $k_{cl}.$ Assuming that Notch expression is maintained constant ( $N_{tot}$ ) the free amount of Notch on the membrane as function of the other species is:

$$Notch_{free}=N_{tot}-N_{C}-N_{N}\cdot \frac{V_{N}}{V_{C}}$$

$N_{C}$ has a molecular weight of 110 kDa, thus it can permeate the nuclear envelope in two modalities: passive and active transport. The former is due to concentration gradient ( $N_{N}-N_{C}$ ) and occurs at rate $k_{tr}^{P}$ . Active transport is modeled with a first-order kinetic (see Supplementary material) with transport rate $k_{\mathrm{tr}}^{A}$ . The differential equations describing $N_{C}$ and $N_{N}$ are:

$$\frac{dN_{C}}{dt}=k_{\mathrm{cl}}\cdot Notch_{free}\cdot D_{E}+\frac{k_{tr}^{P}}{V_{C}}\cdot \left(N_{N}-N_{C}\right)-\frac{k_{\mathrm{tr}}^{A}}{V_{C}}\cdot N_{C}-\mu_{N}\cdot N_{C}$$

$$\frac{dN_{N}}{dt}=\frac{k_{tr}^{P}}{V_{N}}\cdot \left(N_{C}-N_{N}\right)+\frac{k_{\mathrm{tr}}^{A}}{V_{N}}\cdot N_{C}-\mu_{N}\cdot N_{N}$$

$H_{N}^{mRNA}$ represents the concentration of Hes-mRNA in the nucleus, while $H_{C}^{p}$ and $H_{N}^{p}$ are Hes protein concentrations in the cytoplasm and in the nucleus, respectively. We described their dynamics as follows:

$$\frac{dH_{N}^{mRNA}}{dt}=\frac{k_{tH}\cdot N_{N}^{2}}{N_{N}^{2}+K_{H}^{2}}-\mu_{H^{mRNA}}\cdot H_{N}^{mRNA},$$

$$\frac{dH_{C}^{p}}{dt}=k_{H^{p}}\cdot H_{N}^{mRNA}\cdot \frac{V_{N}}{V_{C}}+\frac{k_{tr}^{P}}{V_{C}}\cdot \left(H_{N}^{p}-H_{C}^{p}\right)-\mu_{H^{p}}\cdot H_{C}^{p},$$

$$\frac{dH_{N}^{p}}{dt}=-\frac{k_{tr\;}^{p}}{V_{N}}\cdot \left(H_{N}^{p}-H_{C}^{p}\right)-\mu_{H}^{p}\cdot H_{N}^{p}$$

Transcription of Hes-mRNA was modeled with an activation Hill function $\frac{k_{tH}\cdot N_{N}^{2}}{N_{N}^{2}+K_{H}^{2}}$ , with coefficient equal to 2, and a maximal transcription rate $k_{tH}$ . Hes-mRNA nuclear export was assumed to occur instantaneously and translation into Hes protein occurs at rate $k_{H^{p}}$ . Hes1 has a molecular weight of ∼30 kDa, hence we model nuclear permeation only due to concentration gradient ( $H_{N}^{p}-H_{C}^{p}$ ), with rate $k_{tr}^{P}$ .

Delta transcription is inhibited by $H_{N}^{p}$ , thus we modeled it with a repression Hill function $\frac{k_{tD}\cdot K_{D}^{2}}{K_{D}^{2}+H_{pN}^{2}}$ , with a maximal transcription rate $k_{tD}$ . To simplify our system, we considered Delta mRNA as the final read out, implying that the translation process and protein maturation will simply add a delay to the transcriptional response.

$$\frac{dD}{dt}=\frac{k_{tD}\cdot K_{D}^{2}}{K_{D}^{2}+H_{pN}^{2}}-\mu_{D}\cdot D.$$

All components face degradation, with rates $\mu_{N}$ , $\mu_{H^{mRNA}}$ , $\mu_{H}^{p}$ , and $\mu_{D}$ , where subscripts indicate the respective species.

### Intercellular Signal Communication

The logic formalism was used to describe cell–cell communications during the lateral inhibition process. The epithelium was represented by a two-dimensional grid of cells ( $C_{i}$ ), characterized by the intracellular concentration of Delta (D) binarized accordingly to a fixed threshold ( Th ):

$$C_{i}=\{\begin{array}{cc} 1 & \mathrm{if}\;D\geq Th\\ 0 & \mathrm{if}\;D<Th. \end{array}$$

Delta positive cells influence the neighbors’ fate, activating their Notch pathway. The presence of Delta ligand ( $D_{E}$ ) for the cell $C_{i}$ is a logical function of N neighboring cells.

$$D_{E}\left(C_{1},\ldots ,C_{N}\right)=\{\begin{array}{cc} D_{E}^{\max} & \mathrm{if}\sum_{j=1}^{N}C_{j}^{dist}\geq \vartheta ,\\ 0 & \mathrm{otherwise}. \end{array}$$

where ϑ represents the minimum number of the N neighboring cells required to be Delta positive, and dist indicates the integer distance at which a cell is considered neighbour.

### Model Integration and Simulations

To simulate the pattern development, we integrated the two models. The tissue is represented by a two-dimensional grid of hexagonal cells, each containing the fine-grained kinetic model of Figure 3. Cell–cell communication is instead implemented with logical rules.

To bridge the two representations, for each cell the input of the ODE model ( $D_{E}$ ) is defined according to the state of the neighboring cells via a logical rule. To replicate the salt-and-pepper pattern observed in biological contexts like the *Drosophila* neuronal development, we used the following rule:

$$\begin{array}{c} D_{E}=\{\begin{array}{cc} D_{E}^{\max} & \mathrm{if}\sum_{i=1}^{6}C_{i}^{1}\geq 1,\\ 0 & \mathrm{otherwise}. \end{array} \end{array}$$

**FIGURE 4:**
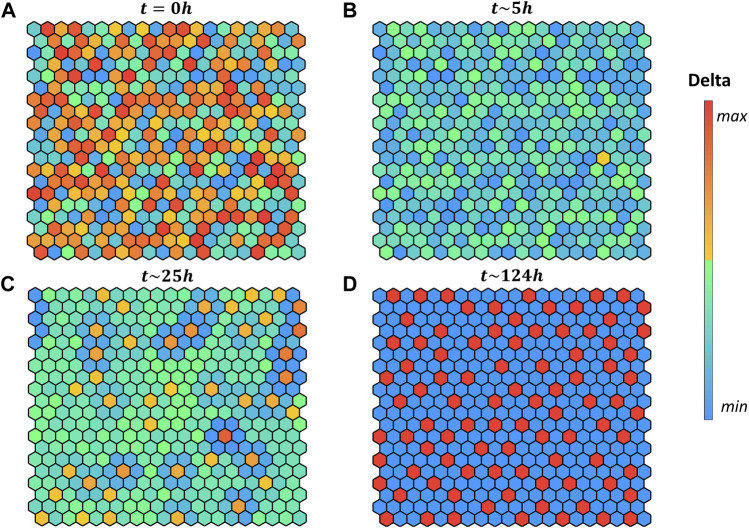
Pattern evolution of a 20 × 20 grid with cylindrical boundary conditions (lateral borders are in contact) using the logical rule $D_{E}=D_{E}^{max}$ if $\sum_{i=1}^{6}C_{i}^{1}\geq 1$ , encoding that the input is present if at least one of the six neighbors at distance one are Delta positive. Colors indicate the intracellular delta concentration according to the color bar. **(A)** Initial values of the variables are set randomly within the biological boundaries. **(B)** All cells in the grid become Delta negative. **(C)** Delta-positive cells start to emerge and affect their neighbour’s fate. **(D)** Equilibrium grid is reached. The hours reported above each grid indicates the quantitative time obtained from the ODE systems.

This implies that a cell receives as input $D_{E}=D_{E}^{max}$ if at least one of the six most proximal neighbors ( dist=1) is delta positive. After initializing the variables of each cell (Figures 4A), the simulation follows the steps described previously until the equilibrium of the grid is reached. To simulate the pattern emergence, we started from random initial conditions, and we selected independently for each cell the species concentrations from their biological range.

Interestingly, in our simulation when starting with random initial conditions (Figures 4A) the grid evolves first toward a naïve state, with no fate decided (Figures 4B), then it converges to the salt-and-pepper pattern accordingly to the selected rule.

**FIGURE 5:**
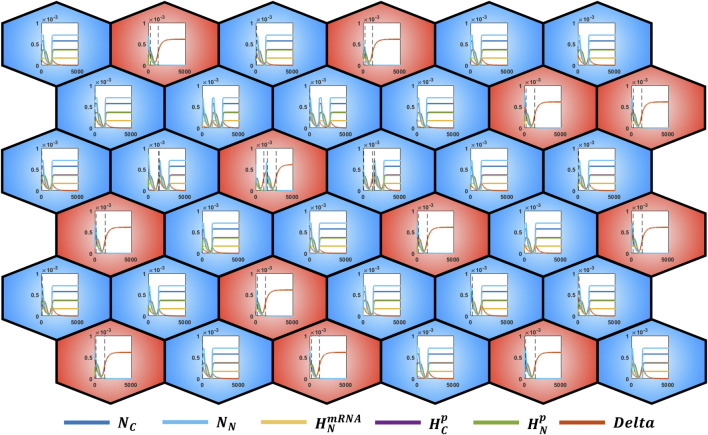
alt-and-pepper pattern of a 6 × 6 grid with cylindrical boundary conditions obtained by applying the rule $D_{E}=D_{E}^{max}$ if $\sum_{i=1}^{6}C_{i}^{1}\geq 2$ . Each cell contains a plot of the computed intracellular variables dynamics; black dashed vertical lines indicate when the cell has generated an event by crossing the Delta threshold. The color of the cell indicates if the dominant variable is Delta or Notch (in agreement with the intracellular dynamics).

Moreover, as illustrated in Figure 5, since the single ODE systems are responsible for the evolution of the epithelial grid, it is possible to observe the dynamics of each module. This approach thus provides multiple levels of information in function of the investigation objectives, allowing to pass from the collective cell behavior to the single intracellular dynamics.

**FIGURE 6:**
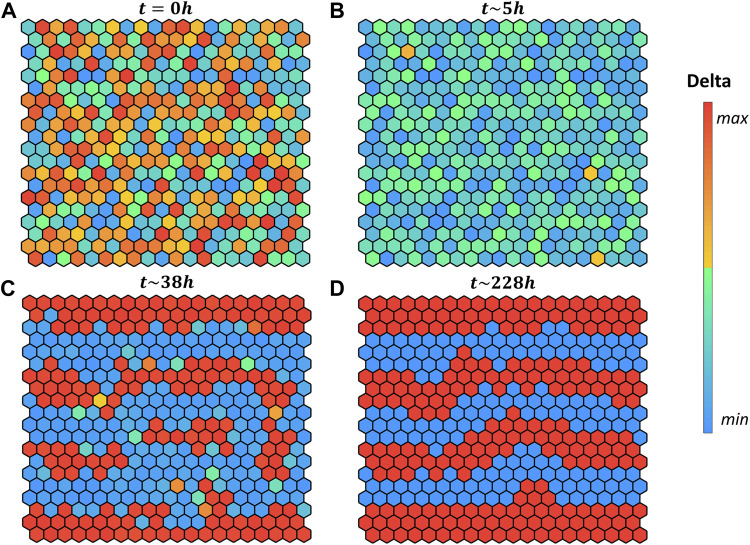
Pattern evolution of a 20 × 20 grid with cylindrical boundary conditions (lateral borders are in contact) using the logical rule $D_{E}=D_{E}^{max}$ if $\sum_{i=1}^{18}C_{i}^{3}\geq 10$ , encoding that the input is present if at least ten neighbors out of the 18 at distance three are Delta positive. Colors indicate the intracellular Delta concentration according to the color bar. **(A)** Initial values of the variables are set randomly within the biological boundaries. **(B)** All cells in the grid become Delta negative. **(C)** Delta-positive cells start to emerge and affect their neighbor’s fate. **(D)** Equilibrium grid is reached. The hours reported above each grid indicates the quantitative time obtained from the ODE systems.

Different rules for $D_{E}$ can include more than one circle of neighbors (distance greater than one), giving rise to a variety of different patterns (Figure 6).

**FIGURE 7:**
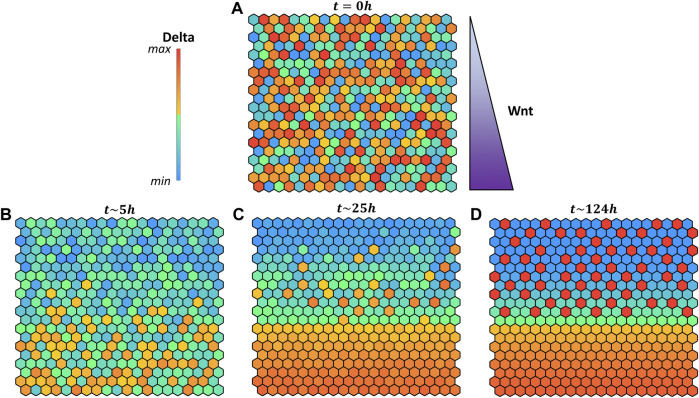
Pattern evolution of a 20 × 20 grid with cylindrical boundary conditions (lateral borders are in contact) using the logical rule $D_{E}=D_{E}^{max}$ if $\sum_{i=1}^{6}C_{i}^{6}\geq 1$ , encoding that the input is present if at least one of the six neighbors at distance one are Delta positive. A positional gradient simulates Wnt concentration over the grid. Colors indicate the intracellular Delta concentration according to the color bar. **(A)** Initial values of the variables are set randomly within the biological boundaries. **(B)** Cells in the upper part of the grid become Delta negative, while on the bottom Wnt inhibition starts to manifest its effect. **(C)** A clear separation between differentiated (Delta + or Delta −) and undifferentiated cells (Wnt) emerges. **(D)** Equilibrium grid is reached. The hours reported above each grid indicates the quantitative time obtained from the ODE systems.

In a cell, several signaling pathways concur to fate decision, in addition to Delta-Notch ( $I_{D}$ ) we can consider the positional input Wnt ( $I_{I}$ ). This soluble protein can affect Notch signaling during fate decisions by diffusing in the tissue and activating a concentration-dependent inhibition of the Notch intracellular domain transcriptional activity (Collu et al., 2012). Wnt was integrated with a fixed concentration dependent on the cell position on the grid (Figure 7). This test case also provides an example of our modeling approach considering an external input not dependent on neighbors.

## Discussion

The description of a multicellular system is always a trade-off between complexity and tractability. Using a fine-grained approach as ODEs (or PDEs) provides a detailed representation of the system, which can be quantitatively used to understand the underpinning mechanism at the core of the biological processes. However, to be able to simulate such models, a large number of parameters are required, eventually leading to parametrization issues. Moreover, moving from a single cell model to a multicellular one will generate a rapid increase in the differential equation number, thus opening the door to integrability and running time problems. On the other hand, parameter-free approaches (such as logic modeling), although able to overcome information gaps, allow only for a qualitative representation of the system behavior. This kind of interpretation can be sometimes insufficient to provide useful insights on biological problems or help to analyze experimental evidence. Here, we introduce a new hybrid approach that leverages on these two formalisms to produce a semi-quantitative representation of a multicellular/tissue environment. Our approach can be also employed to describe interactions among pathways, as bridging signaling cascades by representing the kinase activity as a Boolean variable, or between organs as describing with an ODE system the glucose metabolism and with a logical variable the insulin presence.

To showcase the hybrid formalism, we selected Delta-Notch signaling pathway (Artavanis-Tsakonas et al., 1999) and the consequent cell fate selection in an epithelium (Renaud and Simpson, 2001; De Joussineau et al., 2003). As previously mentioned, different approaches and different granularities have been used to investigate this problem: from coarse-grained intercellular models as the one of Collier et al. (1996) to fine detailed intracellular models as the one of Agrawal et al. (2009). The latter provided an in-depth quantitative description of the Notch signaling pathway, which can be used to investigate the change in phenotypic behavior of the network (from bistable to oscillatory) through sensitivity analysis. However, representing cell–cell communication without embedding the model into a multicellular system may oversimplify important dynamics. Furthermore, fine-grained representation cannot be indefinitely scaled up by simply adding other ODEs for each cell of the epithelium because this would eventually lead to stiffness and numerical instability. The progressively granularity reduction can help to overcome these issues although it provides just a qualitative representation of the system behavior that focuses on the pattern generation: qualitative ODEs (Collier et al., 1996), agent-based systems (Reynolds et al., 2019), and logical models (Varela et al., 2018). These approaches allow to simulate pattern formation at the tissue level but, due to their nature, they either lack quantitative time or information about the species concentrations. To bridge these two levels, we suggest, analogously to hierarchical models (Uluseker et al., 2018), to connect multiple single quantitative modules through the logic formalism. We selected an ODE formalism to describe the intracellular kinetics of the different species which allows to quantitatively trace the system variables for each cell and provide a quantitative time. The rationale to use ODEs for the intracellular pathway hinges on the amount of data and the availability of experimental techniques apt to investigate missing gaps at this level, while cell–cell interaction and tissue dynamics are harder to explore and quantitatively characterize. As represented in Figure 1, each cell can receive two types of input: $I_{D}$ or $I_{I}$ ; the former being used to encode cell–cell communication. The system is simulated following the pseudo-code of Figure 2 until the break condition is met.

In our formalism, the tissue is composed of a grid of cells, each uniquely identified by their position and neighbors. We used a structure made by a 2D single cell layer of hexagonal cells, and the number of neighboring cells varies depending on the distance we consider (6 at distance 1, 12 at distance two etc.). The boundary condition of the grid, important for the evolution, was assumed to be cylindrical (folding the epithelium along the vertical axis) enabling the study of periodic patterns over a larger domain.

The hybrid strategy we propose tries to overcome the conundrum of providing a detailed enough description of the problem while keeping the model complexity under control, each formalism fulfilling a different purpose. The ODE system, being a modular quantitative representation of the intracellular cascade, can be expanded or substituted without major requirements (beside parameter calibration). Furthermore, multicellular logical models of Delta-Notch, as the case study presented by Varela et al. (2018), lack quantitative time and are simulated with a synchronous/asynchronous update of the grid. In our approach, the ODE system provides a quantitative time to the tissue system based on which the cellular grid is updated, allowing for a closer biological interpretation of the resulting dynamics. The internal species dynamics, stored along the simulation, can be instrumental to evaluate the biological processes at different scales.

The logic layer connecting the different single modules is used to describe qualitatively the receptor binding processes between adjacent cells. In addition, it is possible to encode biological information in the system using multilevel inputs; this, together with the logic rule complexity, can account for the receptor binding properties, although at a descriptive level. It is also possible to combine logical dependent inputs, $I_{D}$ , with continuous independent input, $I_{I}$ . This possibility, in the particular case of the Delta-Notch, can be used to model the intestinal crypt, where the soluble factor WNT controls part of the Notch intracellular cascade according to its concentration modulating the cell stemness (Demitrack and Samuelson, 2016). The hybrid strategy can thus be applied to the crypt system, considering Delta as a dependent input coming from the neighboring cells and WNT as continuous input with a concentration gradient (Figure 7).

The hybrid approach we propose in this work, despite the qualitative representation of some model components, can be used to investigate areas in which there are still uncertainties on the underlying mechanism or a lack of the system characterization. This can be used to pave the road toward a more modular representation of biological problems, progressively expanding the current models by replacing the logic parts with more quantitative modules as soon as the necessary information are available.

## Data Availability

The original contributions presented in the study are included in the article/Supplementary Material, further inquiries can be directed to the corresponding author.
