# Supplementary material for: A Novel Hybrid Logic-ODE Modeling Approach to Overcome Knowledge Gaps
Source: Front Mol Biosci. 2021 Dec 20;8:760077. doi: 10.3389/fmolb.2021.760077 (PMC8721169; doi:10.3389/fmolb.2021.760077)
Supplement: Supplementary file 1 [file DataSheet1.pdf]

A novel hybrid logic-ODE modelling approach to overcome knowledge gaps by integrating different granularity approaches.

Gianluca Selvaggio, Serena Cristellon, Luca Marchetti

Supplementary Material

Delta Notch intracellular model

We defined an ODE based model of the Notch signaling cascade following Delta binding. The model is built upon the work of Agrawal et al., (2009) further adding transport mechanisms details and transcriptional processes. Below is reported the full set of ODEs:

$$\frac{dN_C}{dt} = \mathrm{k_{cl}} \cdot Notch_{free} \cdot D_E + \frac{k_{tr}^P}{V_C} \cdot (N_N - N_C) - \frac{\mathrm{k_{tr}^A}}{V_C} \cdot N_C - \mu_N \cdot N_C$$

$$\frac{dN_N}{dt} = \frac{k_{tr}^P}{V_N} \cdot (N_C - N_N) + \frac{\mathrm{k_{tr}^A}}{V_N} \cdot N_C - \mu_N \cdot N_N$$

$$\frac{dH_N^{mRNA}}{dt} = \frac{k_{tH} \cdot N_N^2}{N_N^2 + K_H^2} - \mu_{H^{mRNA}} \cdot H_N^{mRNA},$$

$$\frac{dH_C^p}{dt} = k_{H^p} \cdot H_N^{mRNA} \cdot \frac{V_N}{V_C} + \frac{k_{tr}^P}{V_C} \cdot \left(H_N^p - H_C^p\right) - \mu_{H^p} \cdot H_C^p,$$

$$\frac{dH_N^p}{dt} = -\frac{k_{tr}^p}{V_N} \cdot \left(H_N^p - H_C^p\right) - \mu_{H^p} \cdot H_N^p$$

$$\frac{dD}{dt} = \frac{k_{tD} \cdot K_D^2}{K_D^2 + H_{pN}^2} - \mu_D \cdot \mathrm{D}.$$

$$Notch_{free} = N_{tot} - N_C - N_N \cdot \frac{V_N}{V_C}$$

Model Parametrization

Estimation of translation and transcription rates

To estimate maximal translation rate of Delta (k_{tD}) we used the equilibrium assumption. The external Delta input D_E represents concentration of Delta on the adjacent cells, available for binding with Notch,

and it can be either 0 or D_E^{max}. In absence of Hes, Delta would reach the stable value D_E^{max}. In this case, at equilibrium, maximal translation rate of Delta (k_{tD}) should satisfy the following condition:

$$k_{tD} = \mu_D \cdot D_E^{max}$$

Translation and maximal transcription rates of Hes, as well as the rate of NICD cleavage upon Delta binding, are found in the computational model of Agrawal et al., 2009.

Estimation of degradation rates

For each variable we assumed exponential decay, with constant degradation rates. They are computed from molecule half-life $\tau_{1/2}$, using:

$$\frac{1}{2} = e^{-\mu\tau_{1/2}} \rightarrow \mu = \frac{\ln(2)}{\tau_{1/2}}$$

Ilagan et al. (Ilagan et al., 2011) estimates a half-life of 180 minutes for Notch intracellular domain thus $\mu_{N_C} = \mu_{N_N} = \mu_N = 0.00385$ min^{-1}.

Delta degradation rate is computed from its half-life of 4.9 hr (Preuße et al., 2015). Hes mRNA and protein half-lives are taken from Hirata et al., 2002 and Kobayashi et al., 2015: 24.1 and 22.3 min, respectively.

Transcription processes Hill equations

In any reaction where there is a binding-unbinding process, as per inducible/repressible promoters Hill equations properly depict the complex formation.

$$v = \frac{S^n}{S^n + K_d}$$

Hill equations are characterized by two parameters: K_d or dissociation constant and n the order. The latter usually identify the number of binding sites.

In our model we obtained from literature mining the maximum rate of transcription for Delta and Hes proteins, to represent their transcriptional regulation we multiplied this maximum rate by a Hill equation:

$$\frac{k_{tH} \cdot N_N^2}{N_N^2 + K_H^2}$$

The values for K_H and K_D were selected based upon the maximum concentration of N and H, respectively, to guarantee a full dynamic switch.

Nucleus Import-Export: Passive and Active Transport

Proteins of small dimensions, namely smaller than 60-70 kDa, can move through the nuclear envelope by passive diffusion. Big proteins, on the contrary, require nuclear localization signal (NLS) to be imported into the nucleus. NLS is an aminoacidic sequence which binds to nuclear transport receptors (importins), that mediates the transport of the protein into the nucleus. Nuclear export signal (NES) sequence, analogously, regulates export of a protein, by binding with exportins receptors.

Notch intracellular domain has a molecular weight of 110 kDa (Kelly et al., 2007). It is endowed of nuclear localization signal, while it does not carry NES. Following Cardarelli et al., 2008, which describes transport of a molecule of same size, we model the translocation of NICD in the nucleus, considering passive diffusion and active transport, mediated by NLS, only in entrance.

Table S1: kinetic parameters for the intracellular model, where possible human parameters were preferred.

† Proteomaps, https://www.proteomaps.net/, ‡ used to estimate k_{tr}^{A},[a] assumed, [b] estimated.

Parameter	Description	Value	units	Organism	Ref
k_{tH}	Hes maximal transcription rate	0.0005	μM min^{-1}	Her1 zebrafish	(Agrawal et al., 2009)
k_{tD}	Delta maximal transcription rate	1.4 10^{-6}	μM min^{-1}	-	[b]
k_{cl}	NICD generation constant upon Delta binding	76	μM^{-1} min^{-1}	-	(Agrawal et al., 2009)
V_C	Cytoplasm volume	2.05 10^{-12}	dm^3	HeLa cell human	(Zhao et al., 2008)
V_N	Nuclear volume	3.74 10^{-13}	dm^3	HeLa cell human	(Maul and Deaven, 1977)
K_H	Hill activation coefficient	0.0001	μM	-	[a]
K_D	Hill repression coefficient	0.01	μM	-	[a]
μ_N	NICD degradation rate	0.00385	min^{-1}	Mammals	(Ilagan et al., 2011)
μ_H	Hes mRNA degradation rate	0.029	min^{-1}	Hes1 Mammals	(Hirata et al., 2002)
μ_{H_P}	Hes protein degradation rate	0.032	min^{-1}	Mammals	(Hirata et al., 2002; Kobayashi et al., 2015)

μ_D	Delta degradation rate	0.0024	min^{-1}	DLL1 Mammals	(Preuße et al., 2015)
k_{H_P}	Hes translation rate	4.5	min^{-1}	Vertebrates	(Agrawal et al., 2009)
k_{tr}^{P}	Passive diffusion parameter	4.5 10^{-13}	$dm^3\ min^{-1}$	Chinese hamster	(Cardarelli et al., 2008)
ϕ^M	Michaelis-Menten maximal velocity	8.04 10^{-12}	mol min^{-1}	Mammals	(Cardarelli et al., 2009) ‡
K	Michaelis-Menten coefficient	73.24	μM	Mammals	(Cardarelli et al., 2009) ‡
N_{tot}	Notch cytoplasm concentration	7.06 10^{-4}	μM	HeLa cell human	†
D_{max}^{E}	Jag2 concentration	6.11 10^{-4}	μM	HeLa cell human	†

Hes1 has a size of 280 amino acids and a molecular weight of ~ 30 kDa, being below the threshold mentioned above. Hence, in this case we can consider Hes protein import and export due only to passive diffusion across the nuclear envelope.

Passive diffusion is due to concentration gradients. We used the same parameter k_{tr}^{p}, from Cardarelli et al., 2008, for both NICD and Hes protein.

Active transport was modeled using Michaelis-Menten kinetics. Data from Cardarelli et al., 2009 (Figure S1), expressing flow through the envelope in function of protein concentration were fitted, obtaining maximal velocity ϕ^M and Michaelis-Menten parameter K. Since NICD has concentration at most of order 10^{-3} μ$M \ll K = 73.24$ μM, we can make the following approximation of the Michaelis-Menten term:

$$\frac{\phi^M \cdot N_C}{N_C + K} \sim \frac{\phi^M \cdot N_C}{K} = k_{tr}^{A} \cdot N_C$$

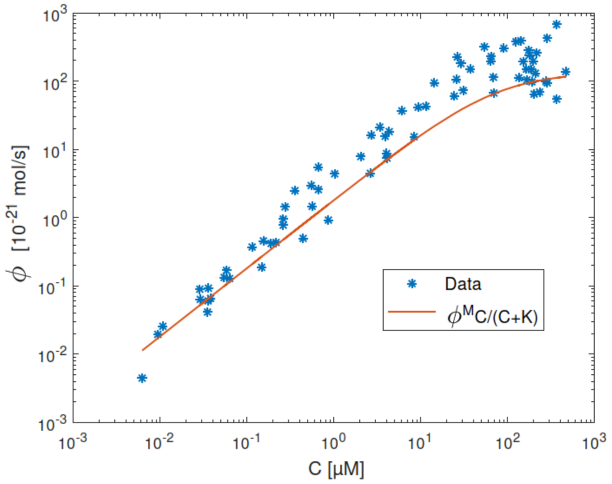

Figure S1: Michaelis-Menten fit of the active import transport.

Estimation of Protein Concentrations Using Proteomic Data

Following Selvaggio et al., 2018 and Milo, 2013 we can compute the concentration of a protein using proteomic abundances, as reported in the repository Proteomaps. This computation assumes, corroborated by evidence, that protein density tends to be constant. For mammalian cells, it is ρ_p= 0.2 g/mL (BNID 105938). If we consider the cell as an envelope containing proteins with constant density, we can thus estimate their average concentration. Given the mean number of amino acids per protein $\overline{L_{aa}}$ = 375 and molecular weight of MW_a = 110 Da per amino acid, we can compute the average concentration of total protein as:

$$C_{tot} = \frac{\rho_p}{fw \cdot MW_a \cdot \overline{L_{aa}}} = 7\ mM,$$

where fw = 0.7 is the fraction of the cell volume occupied by water.

Concentration of a specific protein can be computed as

$$C_{protein} = \frac{\varphi_{protein} \cdot C_{tot}}{\frac{L_{protein}}{\overline{Laa}}}$$

where $\varphi_{protein}$ is the size weighted abundance, expressed in ppm, and $L_{protein}$ is the primary sequence length of the protein.

From Proteomaps (http://www.proteomaps.net) we gather values $\varphi_{Notch} = 0.63 \cdot 10^{-6}$ and $L_{Notch} = 2555$, relative to Notch in the HeLa human cells, leading to a value of Notch cytoplasmatic concentration $N_{tot} = 7.6 \cdot 10^{-4}$ μM. The lack of information on Delta leads us to use another ligand of the same family: Jag2, with $\varphi_{Jag2} = 0.288 \cdot 10^{-6}$ and $L_{Jag2} = 1238$; cell concentration of Jag2 is then $D^{E}_{max} = 6.11 \cdot 10^{-4}$ μM.

Wnt crosstalk

To help understanding the possible application of an independent input we integrated our Delta Notch model with a concentration dependent inhibition of the Notch activity. Due to the lack of experimental data, we used it as a scaling factor that multiplies the rate of Hes-mRNA production

$\frac{dH_N^{mRNA}}{dt} = \frac{K_{Wnt}^2}{Wnt^2+K_{Wnt}^2} \cdot \frac{k_{tH} \cdot N_N^2}{N_N^2+K_H^2} - \mu_{H^{mRNA}} \cdot H_N^{mRNA}$, with the parameter $K_{Wnt} = 0.1\,\mu M$ selected for visualization purposes.

The input Wnt assumes different values, in the interval [0,1], depending only on the cell position in the grid. Starting from the top row where every cell is assigned Wnt=0, then increasing the value stepwise for each row until reaching value Wnt=1 in the cells of the bottom.

Hybrid strategy pseudocode

Below we report the detailed pseudocode of the hybrid strategy simulation algorithm:

Inputs: N ODE-modules with output variable V and independent input (I_I), logical rule (f), break condition, $V_{treshold}$

Output: modules variables time series

while (! Break conditions)

we binarize the grid based on the value of the variable V

$V' \leftarrow V > V_{threshold}$,

we calculate the dependent input I_D applying the logical rule f on a local neighborhood of the binary matrix V'. This is applied on the whole grid

$I_D \leftarrow f(neighbours(V'))$,

we integrate the single modules until they cross the variable V threshold

$Intregrate\ N\ modules\ until\ t_{threshold}, where\ V(i) > V_{threshold}\ i$ ∈ {1:N}

We select the minimum $t_{threshold}$, as it identifies the first update event

$t^{event} \leftarrow \min(threshold)$

We select the module generating the event and select its time variable t to synchronize all the module variables, by interpolation on the same integration steps (variable step algorithms generate different time arrays)

$t \leftarrow Module(t^{event})$

$for\ i = 1{:}N$

$Module(var) \leftarrow interp(Module(var, t))$

end

We then delete all the integration performed after t^{event}

$for\ i = 1{:}N$

$delete\ Module\ variables(t > t^{event})$

end

We then restart the process

end

Per each iteration a single event is generated with a consequent update of the grid. The module variables are always synchronized to the first event in order to generate an output in which all the variables share the same time points for the integration.

References

Agrawal, S., Archer, C., and Schaffer, D. V. (2009). Computational Models of the Notch Network Elucidate Mechanisms of Context-dependent Signaling. *PLoS Comput. Biol.* 5, e1000390. doi:10.1371/journal.pcbi.1000390.

Cardarelli, F., Bizzarri, R., Serresi, M., Albertazzi, L., and Beltram, F. (2009). Probing nuclear localization signal-importin α binding equilibria in living cells. *J. Biol. Chem.* 284, 36638–36646. doi:10.1074/jbc.M109.036699.

Cardarelli, F., Serresi, M., Bizzarri, R., and Beltram, F. (2008). Tuning the transport properties of HIV-1 Tat arginine-rich motif in living cells. *Traffic* 9, 528–539. doi:10.1111/j.1600-0854.2007.00696.x.

Hirata, H., Yoshiura, S., Ohtsuka, T., Bessho, Y., Harada, T., Yoshikawa, K., et al. (2002). Oscillatory expression of the BHLH factor Hes1 regulated by a negative feedback loop. *Science (80-.).* 298, 840–

843. doi:10.1126/science.1074560.

Ilagan, M. X. G., Lim, S., Fulbright, M., Piwnica-Worms, D., and Kopan, R. (2011). Real-time imaging of Notch activation with a luciferase complementation-based reporter. *Sci. Signal.* 4, rs7. doi:10.1126/scisignal.2001656.

Kelly, D. F., Lake, R. J., Walz, T., and Artavanis-Tsakonas, S. (2007). Conformational variability of the intracellular domain of Drosophila Notch and its interaction with Suppressor of Hairless. *Proc. Natl. Acad. Sci. U. S. A.* 104, 9591–9596. doi:10.1073/pnas.0702887104.

Kobayashi, T., Iwamoto, Y., Takashima, K., Isomura, A., Kosodo, Y., Kawakami, K., et al. (2015). Deubiquitinating enzymes regulate Hes1 stability and neuronal differentiation. *FEBS J.* 282, 2475–2487. doi:10.1111/febs.13290.

Maul, G. G., and Deaven, L. (1977). Quantitative determination of nuclear pore complexes in cycling cells with differing DNA content. *J. Cell Biol.* 73, 748–760. doi:10.1083/jcb.73.3.748.

Milo, R. (2013). What is the total number of protein molecules per cell volume? A call to rethink some published values. *BioEssays* 35, 1050–1055. doi:10.1002/bies.201300066.

Preuße, K., Tveriakhina, L., Schuster-Gossler, K., Gaspar, C., Rosa, A. I., Henrique, D., et al. (2015). Context-Dependent Functional Divergence of the Notch Ligands DLL1 and DLL4 In Vivo. *PLoS Genet.* 11. doi:10.1371/journal.pgen.1005328.

Selvaggio, G., Coelho, P. M. B. M., and Salvador, A. (2018). Mapping the phenotypic repertoire of the cytoplasmic 2-Cys peroxiredoxin – Thioredoxin system. 1. Understanding commonalities and differences among cell types. *Redox Biol.* 15, 297–315. doi:10.1016/j.redox.2017.12.008.

Zhao, L., Kroenke, C. D., Song, J., Piwnica-Worms, D., Ackerman, J. J. H., and Neil, J. J. (2008). Intracellular water-specific MR of microbead-adherent cells: The HeLa cell intracellular water exchange lifetime. *NMR Biomed.* 21, 159–164. doi:10.1002/nbm.1173.
