# Supplementary material for: A Novel Hybrid Logic-ODE Modeling Approach to Overcome Knowledge Gaps
Source: Front Mol Biosci. 2021 Dec 20;8:760077. doi: 10.3389/fmolb.2021.760077 (PMC8721169; doi:10.3389/fmolb.2021.760077)
Supplement: Supplementary file 2 [file DataSheet2.ZIP › SelvaggioEtAl_supplementaryMaterial_S2/Readme.docx]

# **A novel hybrid logic-ODE modelling approach to overcome knowledge gaps by integrating different granularity approaches.**

Gianluca Selvaggio, Serena Cristellon, Luca Marchetti

# Supplementary Material

Matlab code to reproduce the results presented in the paper

**IMPORTANT:** the code herein provided is distributed according to the Cosbi Shared Source license agreement ("COSBI-SSLA"). Please, refer to the documentation available at <http://www.cosbi.eu> for any detail.

Content of the folder:

Main_Events: Main file for running simulation, to replicate figure comment rows from 19 to 24.

vicini: Returns a matrix in which rows indicate the six contact neighbours of every cell in the grid

vicini_2: compute neighbours of second order for every cells

vicini_3: compute neighbours of third order for every cells

Parameters_ODE: values of the parameters

rule: compute inputs received by every cell, based on the chosen rule given in the function argument

event_fcn: event location function: event when Delta concentration cross the threshold

integrate_cell: solve ODE system with ode15s

DeltaNotch: system of the ordinary differential equations

hexagon_grid: Draws and colours an hexagonal grid of I rows and J columns

plots: Plots a column of matrix D4 with colormap mymap and a column matrix D3 with mymap2 at every click of the slider

**To re-create some of the Delta-Notch pattern we can run the file: Main_Events.m, after selecting:**

- **The circles of neighbours to be considered in the rule (line 17-32), for figure 4 we select Reg=’1’.**
- **The number of neighbours Delta+ to be Delta-, for figure 4 we considered Rule1 = 1.**

**The output plot we obtained has on the left the current state of the grid, in terms of Delta+/- and on the right the input conditions for each cell.**
